# Dysregulation of Oral Microbial Eicosapentaenoic Acid Induced by Chronic Restraint Stress Exacerbates Periodontitis via M1 Macrophage Polarization

**DOI:** 10.1002/advs.202521346

**Published:** 2026-03-05

**Authors:** Shihong Luo, Fangzhi Lou, Peiran Yang, Yu Zhang, Li Yan, Yunmei Dong, Bing Yang, Haiyang Wang, Yiyun Liu, Juncai Pu, Richard D. Cannon, Peng Xie, Ping Ji, Xin Jin

**Affiliations:** ^1^ The Affiliated Stomatological Hospital Chongqing Medical University Chongqing China; ^2^ The Affiliated Stomatological Hospital Southwest Medical University Luzhou China; ^3^ Chongqing Key Laboratory of Oral Diseases Chongqing China; ^4^ College of Artificial Intelligence Medicine Chongqing Medical University Chongqing China; ^5^ NHC Key Laboratory of Diagnosis and Treatment on Brain Functional Diseases The First Affiliated Hospital of Chongqing Medical University Chongqing China; ^6^ Department of Oral Sciences Faculty of Dentistry Sir John Walsh Research Institute University of Otago Dunedin New Zealand

**Keywords:** chronic restraint stress, eicosapentaenoic acid, macrophages, oral microbiota, periodontitis

## Abstract

The intricate interplay between chronic psychological stress and periodontitis, mediated by oral microbiota and macrophage polarization, remains largely enigmatic. Here, we demonstrate that chronic restraint stress (CRS) exacerbates periodontitis by inducing oral microbial dysbiosis and a consequential shift in host metabolism. Clinical observations reveal a significant correlation between depressive symptoms and the severity of periodontitis, which is underpinned by a distinct oral microbiome. Crucially, fecal microbiota transplantation from CRS‐exposed mice into germ‐free mice was sufficient to transmit the heightened periodontitis phenotype, establishing a causal role for the stress‐altered microbiota. Metabolomic profiling identified a depletion of eicosapentaenoic acid (EPA) in stressed, ligature‐induced periodontitis mice. Mechanistically, supplementation with EPA ameliorates periodontitis by suppressing the NF‐κB signaling pathway, thereby inhibiting the pro‐inflammatory M1 polarization of macrophages. Our findings unveil a novel gut‐oral axis mediated by microbiota and metabolites under stress, and position the omega‐3 fatty acid EPA as a promising therapeutic agent for mitigating stress‐aggravated inflammatory disorders.

## Graphical abstract

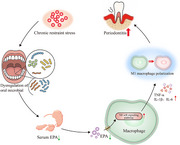

## 1 Introduction

Periodontitis is a prevalent oral disease globally, impacting the oral health of over 30% of adults [1, 2]. It is not only a primary contributor to tooth loss in adults but is also linked to cardiovascular disease, diabetes, and a variety of other systemic health issues [3]. Chronic stress poses a significant challenge to contemporary society, impacting individuals' physical and mental well‐being as well as their overall quality of life [4]. Prolonged stress leads to persistent activation of the body's stress response system, resulting in various physical and mental conditions, including depression [5, 6, 7]. A positive association has been identified between chronic stress and periodontitis [8]. A deeper understanding of the effect of chronic stress on periodontal health is essential for the prevention and treatment of periodontitis. However, studies to date have primarily been clinical epidemiological analyses [9, 10]; the detailed mechanism by which chronic stress promotes periodontitis progression, and potential intervention strategies, remain to be discovered.

Recent findings indicate that oral microbiota dysbiosis contributes significantly to periodontitis by inducing inflammation and disrupting the host‐microbe balance [11, 12]. Our previous studies utilizing a chronic restraint stress (CRS) mouse model revealed that chronic stress can disrupt oral microbiota homeostasis [13]. Accumulating evidence indicates that dysregulation of oral microbiota communities not only orchestrates local inflammatory cascades through microbial‐associated molecular patterns (MAMPs) [14] but also disrupts systemic metabolic regulation via modulation of short‐chain fatty acid levels [15, 16]. Thereby driving the initiation and pathogenesis of periodontitis through synergistic host‐microbe crosstalk [17]. However, whether CRS promotes periodontitis progression by inducing oral microbiota dysbiosis is unknown, and if it does, the underlying mechanism remains unclear.

This study established a clinical cohort of periodontitis patients and developed chronic stress‐periodontitis models in both specific pathogen‐free (SPF) and germ‐free (GF) mice. It used these to investigate whether chronic stress exacerbates periodontitis by modulating the oral microbiota and related metabolites, as well as to explore the underlying mechanisms.

## 2 Results

### 2.1 Association of Depressive Symptoms with Periodontitis Severity and Oral Microbiota Dysbiosis Exacerbation

**FIGURE 1:**
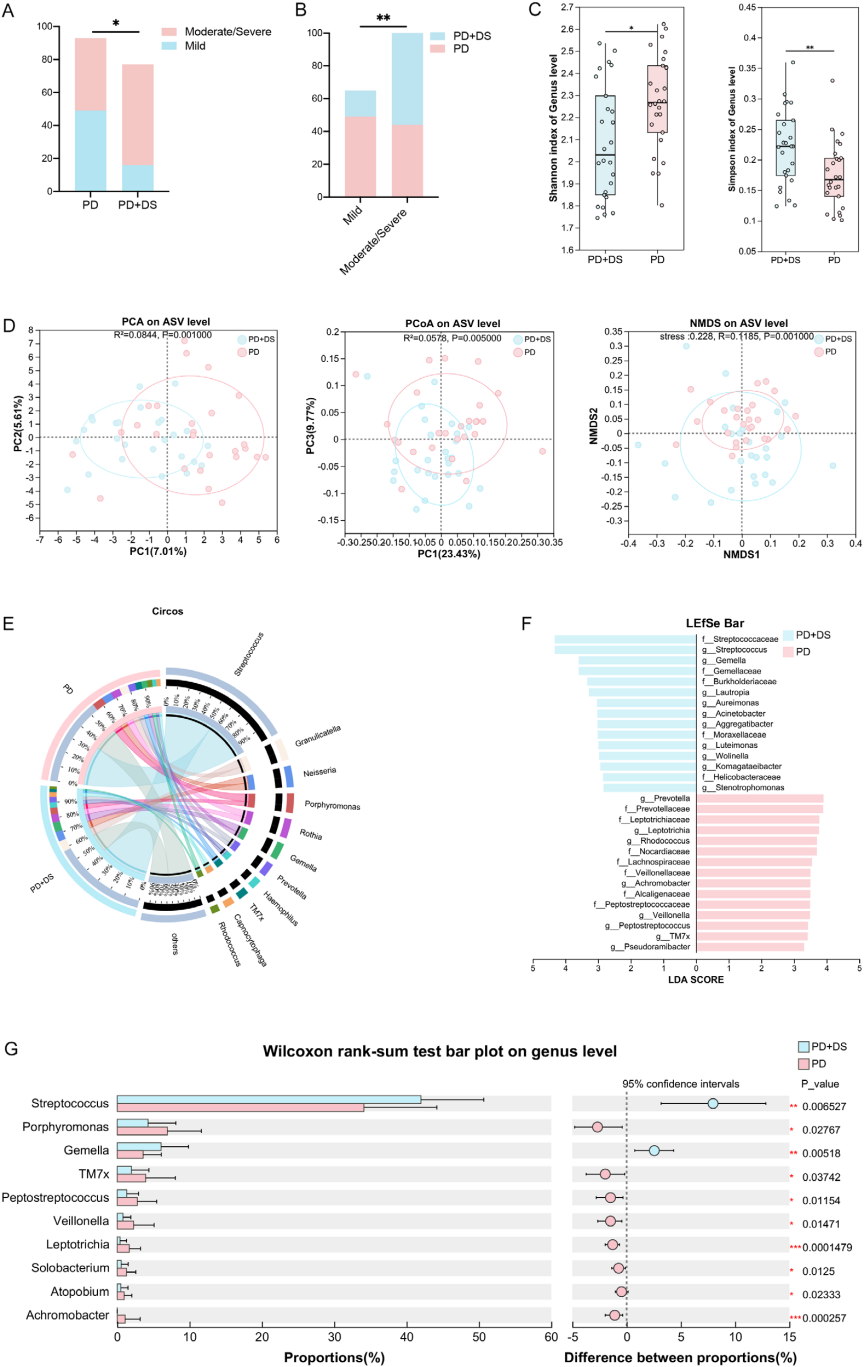
Association between depressive symptoms and periodontitis severity with oral microbiota dysbiosis. (A) The PD group showed balanced mild (53%, 49/93) and moderate/severe (47%, 44/93) periodontitis, while the PD+DS group predominantly had moderate/severe periodontitis (79%, 61/77). (B) Mild periodontitis correlated with lower depression rates (25%, 16/65), whereas the moderate/severe cases showed higher depression prevalence (58%, 61/105). (C) Comparison of alpha diversity indices (Shannon and Simpson) between the PD group and PD+DS group. (D) Beta diversity analysis (PCA, PCoA, and NMDS) was conducted to compare the PD group and the PD + DS group, the significance of beta diversity was assessed using ANOSIM. (E) A Circos plot was used to illustrate the distribution of microbial species at the genus level in the PD and the PD + DS groups. (F) A LEfSe bar chart was employed to identify the microbial taxa that are characteristic of each group. (G) Bacterial genera obtained from oral swabs that exhibited the most significant changes in abundance between the PD group and the PD + DS group, along with their relative abundances. C‐G (PD+DS group, *n* = 30; PD group, *n* = 30). PD, patients with periodontitis and HAMD scores ≤ 7; PD+DS, patients with periodontitis and HAMD scores≥ 8; PCA, principal component analysis; PCoA, principal coordinates analysis; NMDS, non‐metric multidimensional scaling analysis; LEfSe, linear discriminant analysis effect size. ^*^ *p* < 0.05, ^**^ *p* < 0.01, ^***^ *p* < 0.001.

A total of 170 patients with periodontitis were enrolled in this study. The participants' ages varied from 21 to 59, with a median age of 48 (Table 1). To evaluate the association between depressive symptoms and the severity of periodontitis, patients with periodontitis were stratified into two groups based on HAMD scores (PD = 93, PD + DS = 77). Among the PD patients, 49 cases (53%) exhibited mild periodontitis, while 44 (47%) were classified as having moderate/severe periodontitis. In contrast, the PD + DS group contained only 16 mild periodontitis cases (21%), compared with 61 cases (79%) of moderate/severe periodontitis, demonstrating a significantly higher disease severity in this cohort (Figure 1A). From the total number of patients with mild periodontitis (65), 49 (75%) did not experience depression, whereas 16 (25%) displayed depressive symptoms. For those diagnosed with moderate to severe periodontitis (105), it was noted that 44 individuals (42%) did not report depression, while the remaining 61 individuals (58%) did exhibit depressive symptoms (Figure 1B). Pearson correlation tests were conducted on CPI scores and HAMD scores for periodontal status. The correlation coefficient for the correlation test was r = 0.749, with significance (two‐tailed) of *p* < 0.001. These results demonstrate that depressive symptoms are associated with the severity of periodontitis in the clinical cohort.

**TABLE 1:** Basic clinical information for participants in the study.

		Periodontitis (*n* = 170)
Age, mean (SD)		45.5 (9.39)
Gender	male n (%)	48 (28.24)
	female n (%)	122 (71.76)
CPI, mean (SD)		2.67 (0.61)
HAMD, mean (SD)		6.65 (4.26)

To explore the association between depressive symptoms and the oral microbiota in patients with periodontitis, we collected saliva samples from the PD and PD + DS groups for 16S rRNA gene sequencing (*n* = 30 in the PD group; *n* = 30 in the PD + DS group). There were no significant differences in the age (*p* = 0.850), gender (*p* > 0.999), or CPI (*p* = 0.917) between the two groups (Table 2). 16S rRNA gene sequencing analysis showed that there were significant differences in the oral microbial community diversity (Shannon and Simpson index) between the two groups (Figure 1C). Principal component analysis (PCA), principal coordinates analysis (PCoA), and non‐metric multidimensional scaling (NMDS) analyses (beta diversity) revealed a significant segregation in the oral microbiota composition between the two groups (PD+DS vs. PD, *p* < 0.05, ANOSIM) (Figure 1D). Community analysis also confirmed the distinct oral microbial communities in the two groups (Figure 1E). Linear discriminant analysis effect size (LEfSe) analysis showed that the PD+DS group was enriched in the genera *Streptococcus, Gemella*, and *Lautropia* relative to the PD group (Figure 1F). Compared with the PD group, there were 10 bacterial genera the abundances of which were altered significantly (*p* < 0.05, FC > 1.5) in the PD+DS group. Among these, the *Streptococcus* and *Gemella* genera were enriched while oral bacteria in the genera *Porphyromonas*, *TM7x*, and *Peptostreptococcus* were depleted (Figure 1G). These results demonstrate that patients with periodontitis accompanied by depressive symptoms exhibit oral microbiota dysbiosis.

**TABLE 2:** Basic clinical information of salivary 16S rRNA participants.

		PD(*n* = 30)	PD+DS (*n* = 30)	*p*
Age, mean (SD)		49.1 (8.38)	48.7 (7.89)	0.850
Gender	male n (%)	8 (26.67)	8 (26.67)	0.999
	female n (%)	22 (73.33)	22 (73.33)	
CPI, mean (SD)		3.1 (0.25)	3.1 (0.30)	0.917
HAMD, mean (SD)		1.2 (1.01)	11.8 (2.32)	< 0.001

### 2.2 Chronic Restraint Stress‐Induced and Exacerbated Periodontitis

**FIGURE 2:**
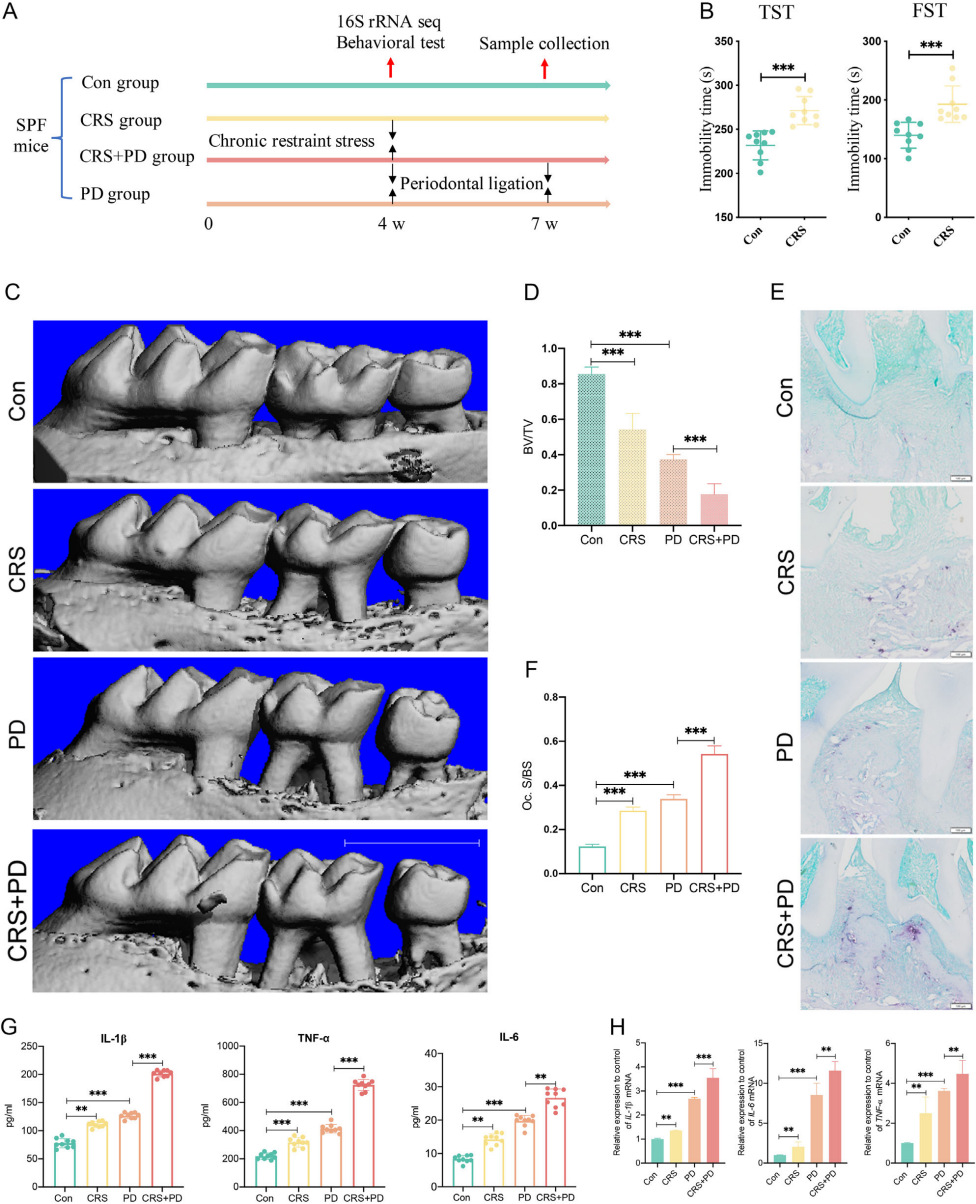
CRS‐induced and exacerbated periodontitis in SPF Kunming mice (*n* = 9 for all groups). (A) Schematic of experimental design assessing the effects of CRS on periodontitis progression and oral microbiome dynamics in mice. (B) CRS‐induced behavioral changes were assessed by immobility duration in the TST and FST assays using EthoVision XT 13.0. (C, D) Micro‐CT analysis of maxillary bone loss: 3D reconstructions (C) and bone volume fraction (BV/TV, %) quantification (D), scale bar = 1 mm. (E, F) Osteoclast activity analysis: TRAP‐stained sections (E) and TRAP^+^ cells/mm^2^ quantification (F). Scale bar = 100 µm. (G) Expression of systemic inflammation markers: IL‐1β, TNF‐α, and IL‐6 serum levels (ELISA). (H) Local cytokine mRNA expression in maxillary gingiva: IL‐1β, IL‐6, TNF‐α (qPCR). Con, control; CRS, chronic restraint stress; PD, periodontal ligation; CRS+PD, chronic restraint stress combined with periodontal ligation; TST, tail suspension tests; FST, forced swim tests. ^*^ *p* < 0.05, ^**^ *p* < 0.01, ^***^ *p* < 0.001.

To investigate the impact of chronic stress on the progression of periodontitis in vivo, we developed a mouse periodontitis model by applying periodontal ligation under CRS conditions. After 4 weeks of CRS, behavioral tests were conducted on mice from both the Con and CRS groups (Figure 2A). We found that the CRS group had a longer total resting time in the TST and FST assays compared to the Con group, suggesting depression‐like behaviors (Figure 2B). Micro‐CT scans of the maxilla revealed that alveolar bone loss was more severe in the CRS group than in the Con group. Furthermore, alveolar bone resorption was even more pronounced in the PD+CRS group than in the PD group (Figure 2C,D). TRAP staining demonstrated an increased number of TRAP‐positive cells in the alveolar bone of mice in the CRS group compared to the Con group. Similarly, the PD+CRS group exhibited a wider distribution of TRAP‐positive cells in the alveolar bone than the PD group (Figure 2E,F). Plasma ELISA results indicated that the levels of inflammatory cytokines IL‐1β, TNF‐α, and IL‐6 were significantly higher in the CRS group than the Con group. Furthermore, the PD + CRS group exhibited elevated levels of IL‐1β, TNF‐α, and IL‐6 relative to the PD group (Figure 2G). In addition, the expression of inflammatory factors in periodontal tissues was markedly increased in the CRS group compared to the Con group. Similarly, the expressions of IL‐1β, IL‐6, and TNF‐α in periodontal tissues were significantly upregulated in the PD + CRS group compared to the PD group (Figure 2H). These results indicate that CRS promotes the progression of periodontitis in mice.

### 2.3 Chronic Restraint Stress Induces Dysbiosis of the Oral Microbiota

**FIGURE 3:**
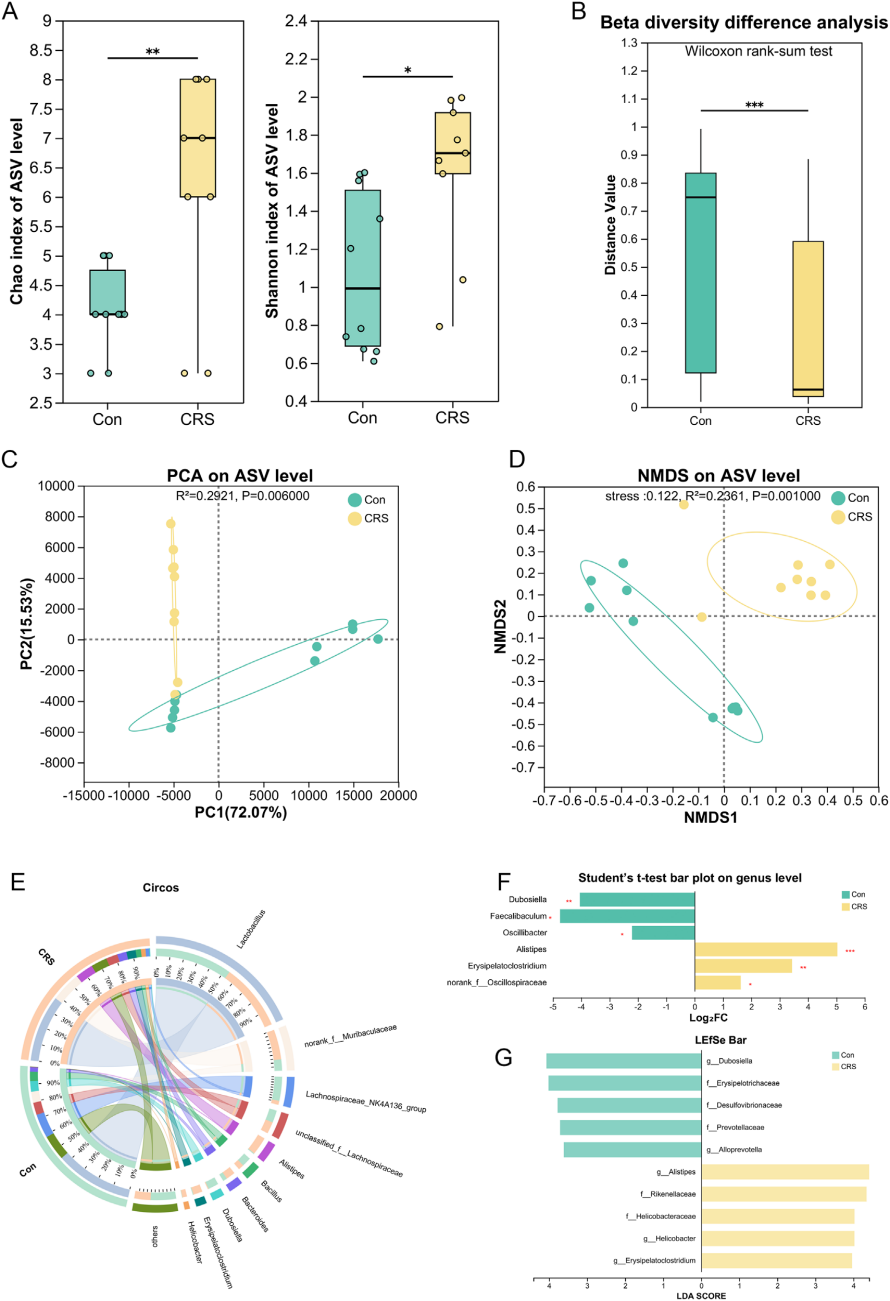
CRS induces oral microbiota dysbiosis in SPF Kunming mice (*n* = 9 for all groups). (A) Comparison of alpha diversity (Chao and Shannon indices) in the Con and CRS groups. (B) Beta‐diversity analysis revealed significant differences in the oral microbial communities of the two groups. (C,D) PCA and NMDS were performed at the ASV level for the Con and CRS groups. (E) Microbial community structure at the genus level visualized through a Circos plot of the Con and CRS groups. (F) Differential abundance analysis of oral microbiota: Significantly altered genera (DESeq2, FDR <0.05) with relative abundance (%) between groups. (G) LEfSe biomarker discovery: Enriched taxa with LDA scores >3.5 distinguishing Con from CRS microbiomes. Con, control; CRS, chronic restraint stress; PCA, principal component analysis; NMDS, non‐metric multidimensional scaling analysis; LEfSe, linear discriminant analysis effect size; LDA, linear discriminant analysis. ^*^ *p* < 0.05, ^**^ *p* < 0.01, ^***^ *p* < 0.001.

To examine the effects of chronic stress on the oral microbiota in mice, we conducted 16S rRNA gene sequencing of salivary samples from the Con group and the CRS group. Alpha diversity (Chao and Shannon index) revealed that the community diversity was significantly higher in the CRS group compared to the Con group (Figure 3A). PCA and NMDS analyses (Beta diversity analysis) demonstrated distinct clustering within groups and clear separation between the Con and CRS groups (Figure 3B–D). In addition, the composition of oral microbiota differed between the CRS and Con groups (Figure 3E). At the genus level, *Alistipes*, *Erysipelatoclostridium*, and *Oscillospiraceae* were found to be more prevalent and *Dubosiella, Faecalibaculum*, and *Oscillibacter* less prevalent in the CRS mice than in the Con mice (Figure 3F). LEfSe analysis showed that the CRS group was enriched in *Alistipes*, *Rikenellaceae*, and *Helicobacteraceae* compared with the Con group (Figure 3G). These results indicate that CRS alters the oral microbiota composition in mice.

### 2.4 Chronic Restraint Stress ‐Induced Microbiota Dysbiosis Exacerbates Periodontitis in Germ‐Free Mice

**FIGURE 4:**
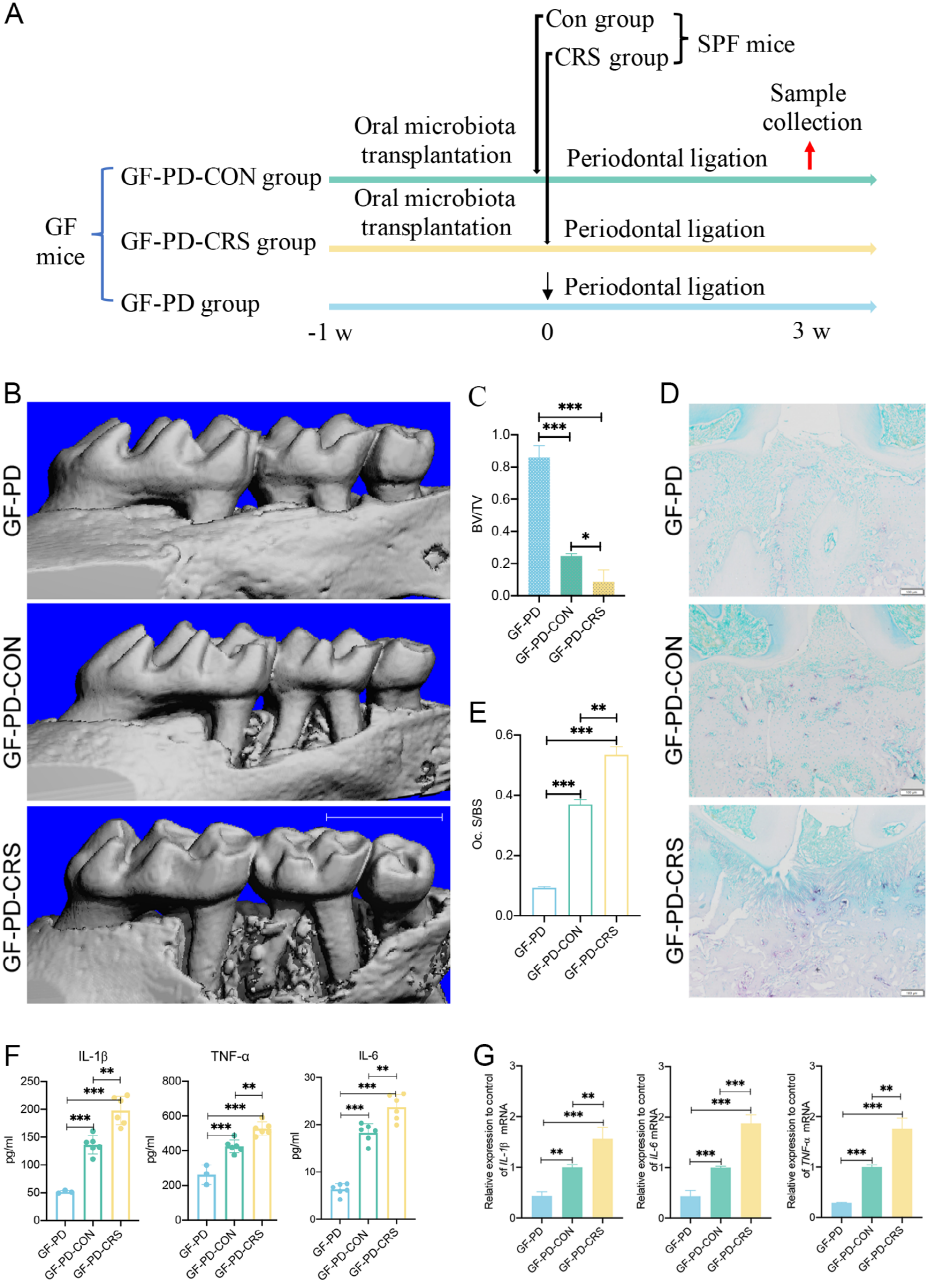
CRS‐induced microbial dysbiosis exacerbates periodontitis progression in germ‐free Kunming mice (GF‐PD group, *n* = 3; GF‐PD‐CON group, *n* = 6; GF‐PD‐CRS group, *n* = 6). (A) Schematic of experimental design of oral microbiota transplantation (OMT) from Con/CRS donors to germ‐free (GF) mice. (B,C) Micro‐CT analysis of maxillary bone loss: 3D reconstructions (B) and bone volume fraction (BV/TV, %) quantification (C) scale bar = 1 mm. (D,E) Osteoclast activity analysis: TRAP^+^ cell visualization in maxillary sections (D) and quantification of osteoclasts/mm^2^ (E). Scale bar = 100 µm. (F) Expression of systemic inflammation markers: IL‐1β, TNF‐α, and IL‐6 serum levels (ELISA). (G) Local osteolytic cytokine expression: IL‐1β/IL‐6/TNF‐α mRNA levels in gingival tissue (qPCR). GF‐PD, GF Kunming mice with periodontal ligation; GF‐PD‐CON, GF Kunming mice with periodontal ligation receiving OMT from Con donors; GF‐PD‐CRS, germ‐free Kunming mice with periodontal ligation receiving OMT from CRS donors. ^*^ *p* < 0.05, ^**^ *p* < 0.01, ^***^ *p* < 0.001.

To verify the direct role of CRS‐altered oral microbiota in periodontitis progression, we transplanted oral microbes from CRS or CON mouse donors to GF mice subjected to periodontal ligation (Figure 4A). Micro‐CT scans revealed that GF mice colonized with oral microbiota from CRS mice (GF‐PD‐CRS) exhibited significantly greater alveolar bone loss than GF mice colonized with oral microbiota from CON mice (GF‐PD‐CON). However, both the GF‐PD‐CRS and GF‐PD‐CON groups showed more alveolar bone loss than GF mice without microbial transplantation (GF‐PD) (Figure 4B,C). TRAP staining demonstrated that the number of TRAP‐positive cells in the alveolar bone was significantly higher in the GF‐PD‐CRS group than in the GF‐PD‐CON group. In addition, both the GF‐PD‐CRS and GF‐PD‐CON groups exhibited larger TRAP‐positive areas than the GF‐PD group (Figure 4D,E). ELISA analysis of plasma inflammatory factors revealed that the concentrations of IL‐1β, TNF‐α, and IL‐6 were significantly elevated in the GF‐PD‐CRS group compared to the GF‐PD‐CON group (Figure 4F). Furthermore, both the GF‐PD‐CRS and GF‐PD‐CON groups exhibited significantly higher concentrations of IL‐1β, IL‐6, and TNF‐α than the GF‐PD group (Figure 4G). These results suggest that CRS‐induced oral microbiota dysbiosis may directly promote periodontitis progression in GF mice.

### 2.5 Dysbiotic Oral Microbiota Caused by Chronic Restraint Stress Modulates the Host Metabolome in Germ‐free Mice

**FIGURE 5:**
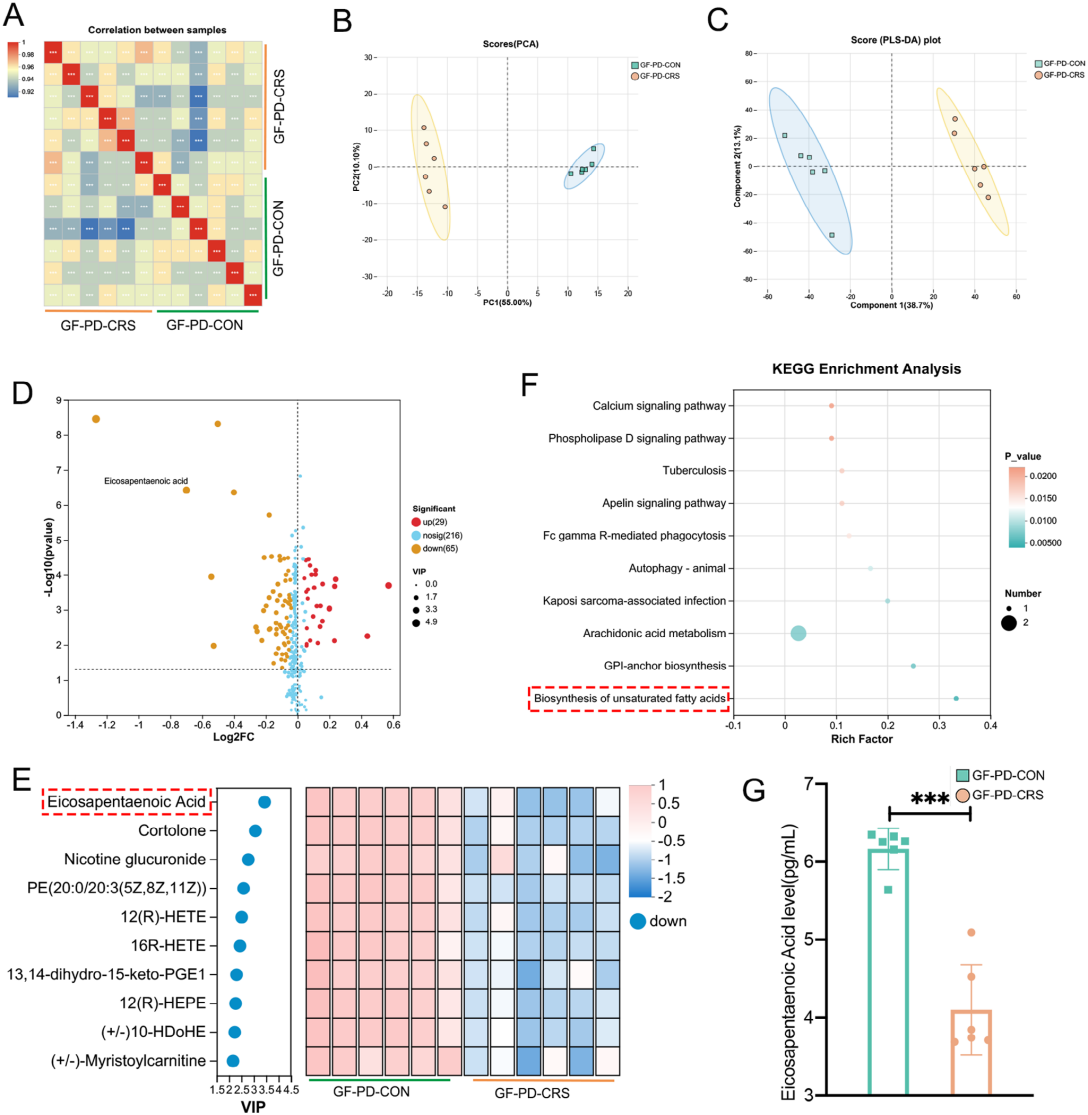
CRS‐induced oral dysbiosis remodels the host metabolome in GF Kunming mice (*n* = 6 for all groups). (A) Plasma metabolic signature stratification: Hierarchically clustered heatmap (Z‐score normalized) of differentially abundant metabolites (VIP > 1.0). (B) PCA revealed a clear separation of plasma metabolite profiles between the two groups. (C) PLS‐DA revealed a clear separation of plasma metabolite profiles between the two groups. (D) Volcano plot illustrating changes in metabolite concentrations in GF‐PD‐CRS mouse plasma relative to GF‐PD‐CON mouse plasma. The *x*‐axis represents log_2_‐transformed FC of metabolite abundances, and the y‐axis represents log_10_‐transformed p‐values determined by the Wilcoxon rank‐sum test. The horizontal line indicates *p* <0.05, and the vertical lines indicate FC thresholds of >1.5 or <0.67. Metabolites with increased or decreased concentrations are highlighted in red or yellow, respectively. (E) Key metabolic discriminators: Top 10 dysregulated metabolites (VIP>2.0, FDR‐adjusted *p* <0.05) distinguishing the GF‐PD‐CRS group from GF‐PD‐CON group, annotated with KEGG compound IDs. Bars indicate log_2_ fold‐changes (two‐tailed Mann‐Whitney U test). (F) PUFA metabolism in ligature‐induced periodontitis mice was significantly influenced by CRS‐altered oral microbiota. (G) The GF‐PD‐CRS group exhibited significantly lower plasma EPA levels than the GF‐PD‐CON group. GF‐PD‐CON, GF Kunming mice with periodontal ligation receiving OMT from Con donors; GF‐PD‐CRS, germ‐free Kunming mice with periodontal ligation receiving OMT from CRS donors; PUFA, Polyunsaturated fatty acid; EPA, eicosapentaenoic acid; PCA, principal component analysis; PLS‐DA, partial least squares‐discriminant analysis; KEGG, Kyoto Encyclopedia of Genes and Genomes; FC, fold change; VIP, variable importance in projection. ^***^ *p* < 0.001.

Next, untargeted metabolomics sequencing was employed to investigate the impact of CRS‐induced oral microbiota alterations on host metabolism in mice with periodontal ligation, with subsequent identification of potential key metabolites. First, based on Bray‐Curtis distance metrics, Pearson's correlation test was employed to assess variations in metabolite composition and abundance between the GF‐PD‐CON and GF‐PD‐CRS groups. Heatmap visualization revealed significant inter‐sample correlations, indicating high similarity in metabolic profiles and abundance patterns among samples (Figure 5A). PCA and PLS‐DA analyses indicated that the samples from each group cluster together, with significant differences between the groups (Figure 5B,C). Second, volcano plot analysis revealed a total of 94 metabolites with significantly altered concentrations between the GF‐PD‐CRS and GF‐PD‐CON groups, comprising 29 upregulated and 65 downregulated metabolites (Figure 5D). Then, we obtained the top 10 differential metabolites through VIP value analysis. The top 10 metabolites with altered abundance included eicosapentaenoic acid (EPA), cortolone, and nicotine glucuronide, which were decreased in the GF‐PD‐CRS mice (Figure 5E). Kyoto Encyclopaedia of Genes and Genomes (KEGG) enrichment analysis of the metabolome data identified 10 pathways that were modified between the GF‐PD‐CRS and GF‐PD‐CON mice. Notably, the polyunsaturated fatty acid (PUFA) metabolism in ligature‐induced periodontitis mice was significantly influenced by CRS‐altered oral microbiota (Figure 5F). As a pivotal member of the ω‐3 fatty acid family, EPA participates in PUFA metabolism. Targeted metabolomics analysis further confirmed that the plasma EPA concentration in the GF‐PD‐CRS group was significantly lower than that in the GF‐PD‐CON group (Figure 5G). These results indicate that oral microbial dysbiosis induced by CRS may affect periodontitis progression by altering the host metabolome.

### 2.6 EPA Attenuates Periodontal Inflammation by Suppressing M1 Macrophage Polarization

**FIGURE 6:**
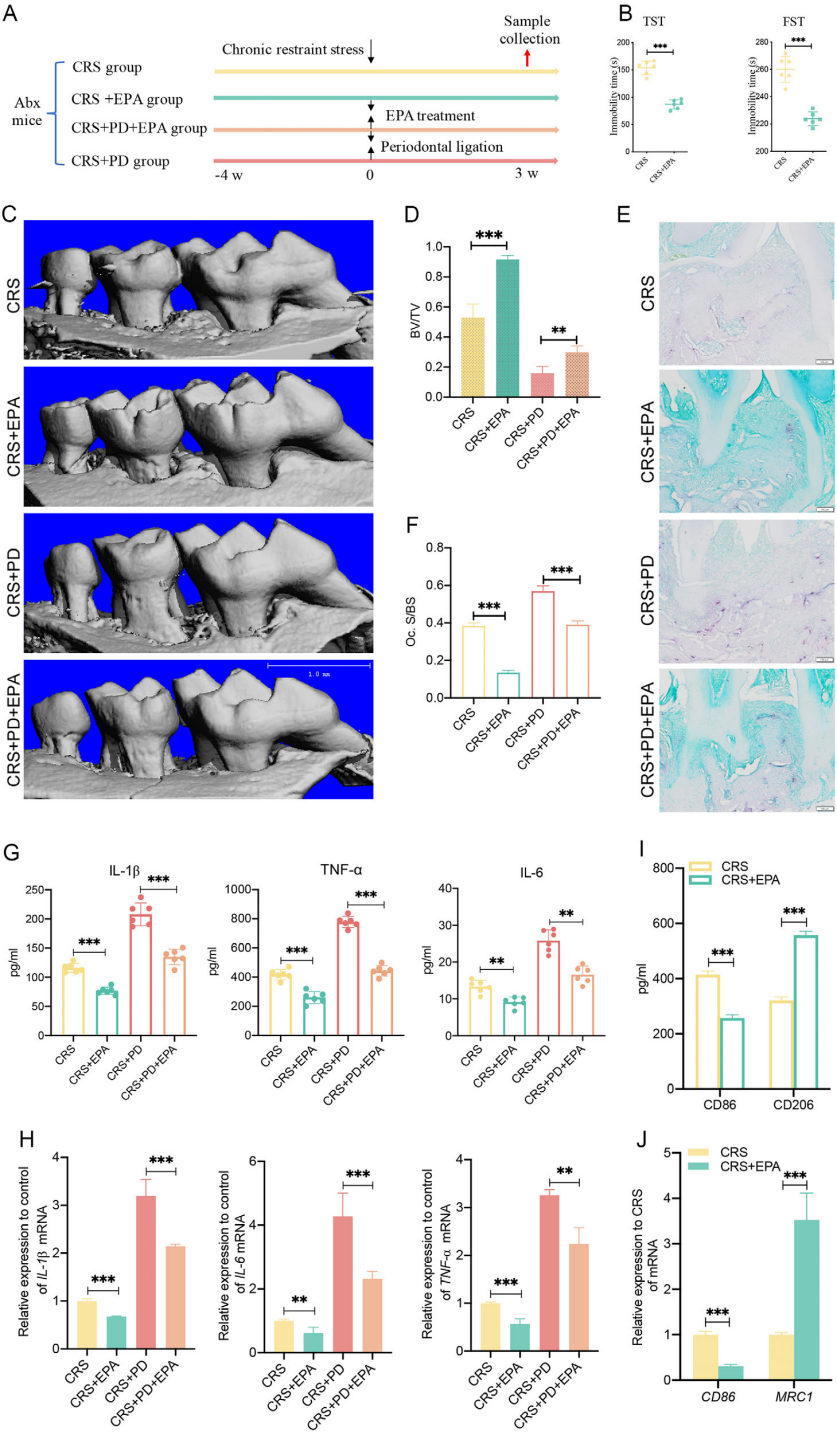
EPA attenuates periodontal inflammation by suppressing M1 macrophage polarization (*n* = 6 for all groups). (A) Schematic of experimental design evaluating the therapeutic efficacy of EPA on periodontitis under chronic stress conditions. (B) Behavioral profiling: EPA treatment (50 mg/kg) significantly reduced depressive‐like behavior in CRS mice, evidenced by TST and FST assays using EthoVision XT 13.0. (C, D) Micro‐CT analysis of maxillary bone loss: 3D reconstructions (C) and bone volume fraction (BV/TV, %) quantification (D). Scale bar = 1 mm. (E,F) Osteoclast activity analysis: TRAP^+^ cell visualization in maxillary sections (E) and quantification of osteoclasts/mm^2^ (F). Scale bar = 100 µm. (G) Expression of systemic inflammation markers: IL‐1β, TNF‐α, and IL‐6 serum levels (ELISA). (H) Local osteolytic cytokine expression: IL‐1β/IL‐6/TNF‐α mRNA levels in gingival tissue (qPCR). (I) ELISA quantification of macrophage polarization markers CD86 (M1) and CD206 (M2) in periodontal tissues. (J) qPCR analysis of macrophage polarization markers CD86 (M1) and MRC1 (M2) in EPA‐treated periodontal tissues. CRS, chronic restraint stress; PD, periodontal ligation; EPA, eicosapentaenoic acid.; TST, tail suspension tests; FST, forced swim tests. ^**^ *p* < 0.01, ^***^ *p* < 0.001.

Subsequently, we investigated the therapeutic potential of the microbial metabolite EPA in periodontitis under CRS conditions. ABX‐treated mice underwent 4 weeks of CRS, with or without a concurrent 3‐week periodontal ligation and EPA treatment. Behavioral tests were performed on the CRS and CRS+EPA groups prior to the collection of biological samples for subsequent analysis (Figure 6A). TST and FST behavioral tests showed that mice in the CRS+EPA group had lower total rest time than the CRS group (Figure 6B). Micro‐CT scans revealed that alveolar bone loss in the CRS+EPA group was lower than in the CRS+PD group, while the remaining alveolar bone mass in the CRS+PD+EPA group was greater than that in the CRS+PD group (Figure 6C,D). TRAP staining results were consistent with those of Micro‐CT scans, suggesting that EPA supplementation inhibited osteoclast activity in alveolar bone (Figure 6E,F). ELISA results indicated that the concentrations of inflammatory factors IL‐1β, TNF‐α, and IL‐6 in the plasma of mice in the CRS+EPA group were lower than those in the CRS group, while the levels of IL‐1β, TNF‐α, and IL‐6 in the CRS+PD+EPA group were also reduced compared to the CRS+PD group (Figure 6G). The qPCR analysis of mRNA levels was consistent with the results obtained from the ELISA assays (Figure 6H).

To explore the mechanism by which EPA ameliorates the progression of periodontitis induced by CRS, ELISA and qPCR were used to assess macrophage polarization markers in periodontal tissues. The ELISA results indicated a reduction in M1 marker CD86 expression and an elevation in M2 marker CD206 levels in the periodontal tissues of the CRS+EPA group compared to the CRS group (Figure 6I). qPCR analysis revealed that CD86 expression was lower while M2 marker MRC1 expression was higher in the periodontal tissues of mice treated with EPA than in the CRS group (Figure 6J). These results suggest that under stress conditions, EPA may alleviate periodontal inflammation, potentially through suppressing macrophage polarization toward the pro‐inflammatory M1 phenotype.

### 2.7 EPA Inhibits M1 Polarization via NF‐κB Pathway Blockade

**FIGURE 7:**
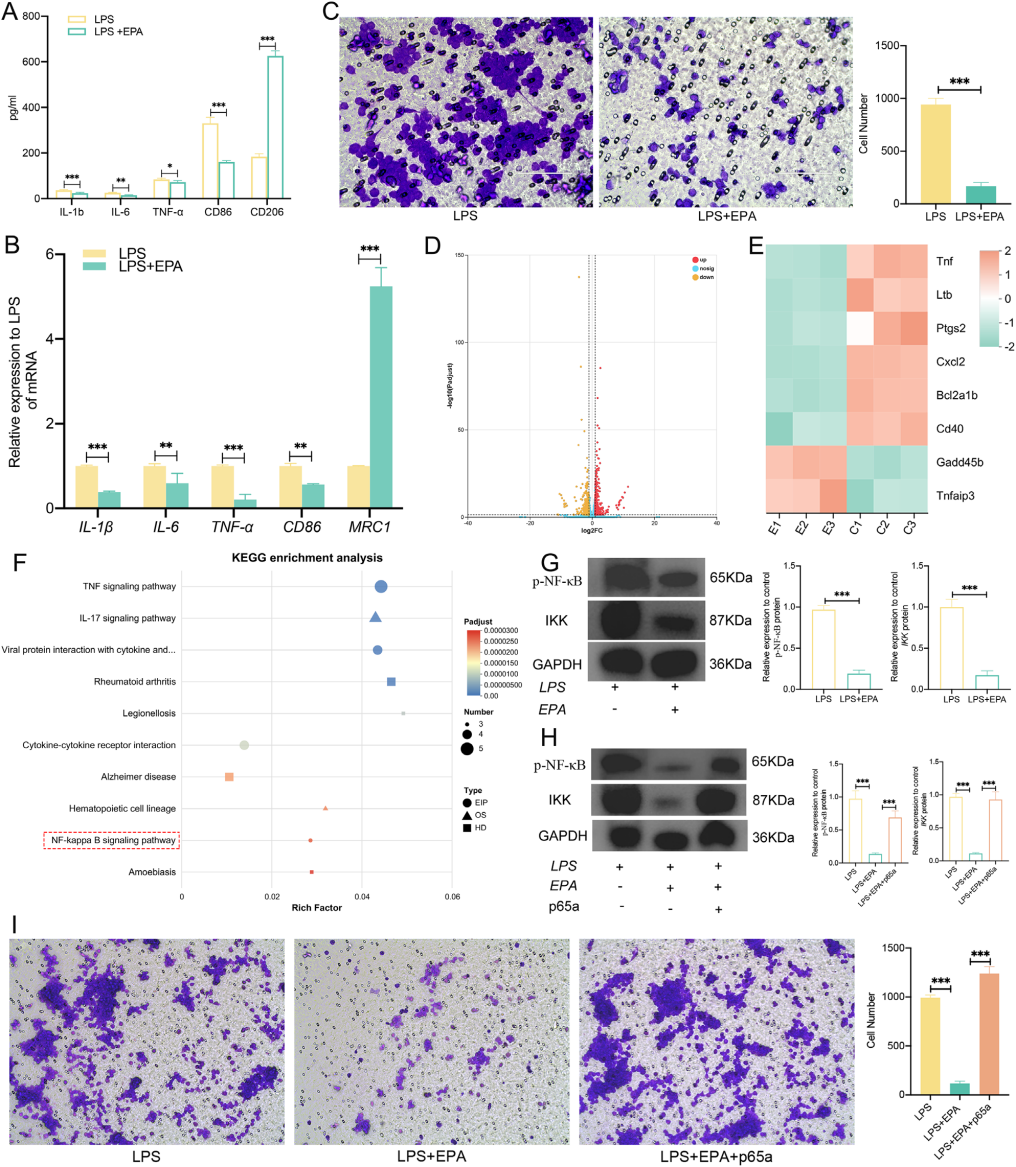
EPA reduces M1 macrophage polarization via the NF‐κB pathway. (A) The concentrations of IL‐1β, TNF‐α, IL‐6, CD86, and CD206 in the culture supernatant of RAW264.7 cells were determined using ELISAs. (B) mRNA expression levels of IL‐1β, TNF‐α, CD86, and MRC1 in RAW264.7 cells were analyzed via qPCR. (C) Representative images of RAW264.7 cell migration in transwell assays and quantification of migrated cells. Scale bar = 100 µm. (D) Volcano plot showing changes in gene expression in EPA‐treated RAW264.7 cells compared to expression in control cells. Differential expression analysis was performed using DESeq2 (based on a negative binomial distribution). Genes with increased or decreased expression are highlighted in red or green, respectively. (E) Heatmap showing the differentially expressed genes in the Con and EPA groups. (F) KEGG functional annotation of differentially expressed genes related to the NF‐κB signaling pathway. (G) Western blot analysis of p‐NF‐κB and IKK protein expression levels in LPS‐stimulated RAW264.7 cells treated with EPA. (H) Western blot analysis of p‐NF‐κB and IKK protein expression levels in LPS‐stimulated RAW264.7 cells treated with EPA or p65a. (I) Representative images of RAW264.7 cell migration in transwell assays and quantification of migrated cells. Scale bar = 400 µm. KEGG, Kyoto Encyclopedia of Genes and Genomes; LPS, lipopolysaccharide; EPA, eicosapentaenoic acid; p65a, the NF‐κB/p65 activator. ^*^ *p* < 0.05, ^**^ *p* < 0.01, ^***^ *p* < 0.001.

We systematically evaluated the effects of EPA on Raw264.7 macrophage polarization in vitro. ELISA assays demonstrated that EPA treatment decreased the levels of IL‐1β, TNF‐α, IL‐6, and CD86 while increasing CD206 levels in Raw264.7 macrophages (Figure 7A). Similarly, qPCR results supported these findings, indicating that EPA effectively inhibited M1‐type polarization in Raw264.7 cells and reduced the inflammatory response (Figure 7B). Furthermore, transwell experiments showed that EPA treatment significantly impaired the migration ability of Raw264.7 cells (Figure 7C). To investigate how EPA influences macrophage polarization, RNA‐seq was performed on macrophages from both the LPS‐CON and LPS‐EPA groups. The results showed that a total of 1,144 genes exhibited significant alterations (a fold change exceeding 2.0 and a p‐value below 0.5) between the two groups. Among these differences in gene expression, the expression of 545 genes was decreased, while the expression of 599 genes was increased (Figure 7D). Notably, the expression of many NF‐κB pathway markers changed in the LPS‐EPA group compared with LPS‐Con group, including Tnf, Ltb, Ptgs2, Cxcl2, Bcl2a1b, Cd40, Gadd45b, and Tnfaip3 (Figure 7E). KEGG enrichment analysis revealed that EPA induced the modulation of the NF‐κB signaling pathway in Raw264.7 cells (Figure 7F). Western blot was used to verify the results of RNA‐seq analysis, and we found that EPA treatment inhibited the expression of p‐NF‐κB and IKK proteins in Raw264.7 cells (Figure 7G).

To investigate whether inhibition of the NF‐κB pathway is the mechanism by which EPA affects the polarization and invasion of macrophages, Raw264.7 cells treated with EPA were subsequently exposed to the NF‐κB/p65 activator (p65a). Western blot analysis indicated that EPA treatment inhibited the expression of p‐NF‐κB and IKK proteins, while the expression of both p‐NF‐κB and IKK proteins increased following p65a treatment (Figure 7H). qPCR results demonstrated that EPA treatment inhibited the expression of IL‐1β, IL‐6, TNF‐α, and CD86. In contrast, the expressions of MRC1 and Arg1 were increased (Figure 1). Transwell assays showed that EPA treatment inhibited Raw264.7 migration, and this effect was reversed by p65a (Figure 7I). These results suggest that EPA reduces M1 macrophage polarization via the NF‐κB pathway.

### 2.8 NF‐κB Activation Abrogates EPA's Benefit in Periodontal Inflammation

**FIGURE 8:**
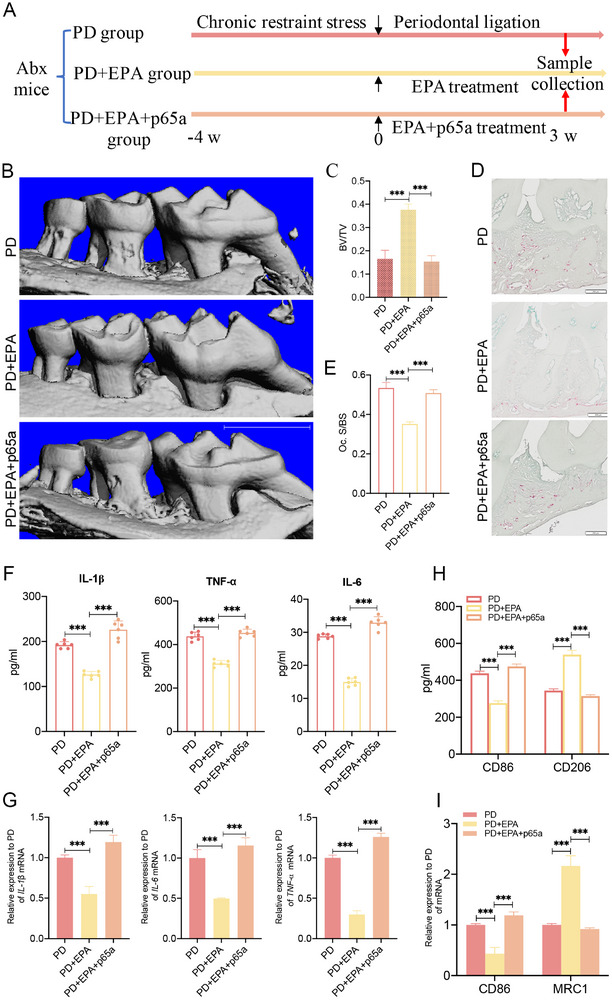
NF‐κB activation abrogates EPA's benefit in periodontal inflammation (*n* = 6 for all groups). (A) Schematic of experimental design evaluating the role of NF‐κB signaling in EPA‐mediated therapy for periodontitis under CRS. (B,C) Micro‐CT analysis of maxillary bone loss: 3D reconstructions (B) and bone volume fraction (BV/TV, %) quantification (C). Scale bar = 1 mm. (D,E) Osteoclast activity analysis: TRAP^+^ cell visualization in maxillary sections (D) and quantification of osteoclasts/mm^2^ (E). Scale bar = 100 µm. (F) Expression of systemic inflammation markers: IL‐1β, TNF‐α, and IL‐6 serum levels (ELISA). (G) Local osteolytic cytokine expression: IL‐1β, IL‐6, and TNF‐α mRNA levels in gingival tissue (qPCR). (H) Protein levels of macrophage polarization markers (CD86/M1, CD206/M2) in periodontal tissues by ELISA. (I) mRNA levels of macrophage polarization markers (CD86/M1, CD206/M2) in periodontal tissues by qPCR. CRS, chronic restraint stress; PD, periodontal ligation; EPA, eicosapentaenoic acid.; p65a, the NF‐κB/p65 activator. ^***^ *p* < 0.001.

To definitively establish the pivotal role of NF‐κB signaling in mediating EPA's therapeutic effects, we employed a comprehensive in vivo model. After ABX and 4 weeks of CRS, mice underwent 3 weeks of periodontal ligation, during which they received EPA, a combination of EPA and p65a, or vehicle control (Figure 8A). Micro‐CT analysis revealed that EPA treatment attenuated alveolar bone loss, but this protective effect was crucially reversed by co‐administration of p65a (Figure 8B,C). Consistently, TRAP staining demonstrated that EPA suppressed osteoclast activity, an effect that was nullified by p65a (Figure 8E,F). At the systemic level, ELISA showed that EPA reduced plasma levels of pro‐inflammatory cytokines (IL‐1β, TNF‐α, and IL‐6), and this anti‐inflammatory action was likewise abrogated by p65a (Figure 8F). These findings were further confirmed at the transcriptional level by qPCR (Figure 8G). Collectively, our results demonstrate that under CRS, the therapeutic benefits of EPA on periodontal inflammation are effectively reversed by NF‐κB pathway activation.

To assess the impact of EPA and p65a on macrophage polarization in periodontal tissues, we analyzed the expression of polarization markers using ELISA and qPCR. ELISA results revealed that, compared to the PD group, EPA treatment (PD+EPA) shifted the macrophage profile toward an M2 phenotype, as evidenced by a decrease in the M1 marker CD86 and an increase in the M2 marker CD206. Conversely, the addition of p65a (PD+EPA+p65a group) reversed this shift, elevating CD86 and reducing CD206 levels relative to the PD+EPA group (Figure 8H). These protein‐level findings were corroborated by qPCR analysis at the mRNA level (Figure 8I), confirming that EPA promotes M2 polarization in a manner reversible by NF‐κB pathway activation.

## 3 Discussion

Previous studies indicate that depressed individuals often experience poor oral health, yet the exact relationship between the two is still unclear [18]. In this study, we found that patients with moderate and severe periodontitis had a higher incidence of depressive symptoms than patients with mild periodontitis. Although the precise biological mechanisms underlying their comorbidity remain incompletely elucidated, systemic inflammation may be a key factor [19, 20]. Microbial imbalance has also been suggested as a potential link between depressive symptoms and periodontitis. The oral microbiota of patients with periodontal disease is significantly different from that of healthy individuals, with an increased number of certain pathogens, which may affect distal organs, including the brain, and may affect emotional regulation through immunomodulatory mechanisms [21]. In this study, 16S rRNA gene sequencing of saliva samples from patients with periodontitis alone and periodontitis accompanied by depressive symptoms revealed a significant dysbiosis in the structure of the oral microbial community.

CRS is commonly used to simulate chronic stress in animal models as it accurately reflects the behavioral symptoms that humans may experience under prolonged stress, such as depression‐like behavior [22]. In addition, studies have shown that CRS leads to changes in various biological processes. These include alterations in neurotransmitter levels and increased neuroinflammation, similar to the biomarker changes seen in human depression [23, 24]. CRS has been shown to not only affect psychological states, such as inducing depression‐like behaviors, but also trigger a series of inflammatory responses in the animals, including exacerbating periodontal inflammation [25]. In the present study, periodontal inflammation was worse in mice with chronic stress than in controls. Chronic stress increases cortisol levels by activating the neuroendocrine system [26]. Elevated levels of cortisol and other stress hormones may directly affect periodontal tissues and aggravate periodontal inflammation [27]. Recently, more and more studies have begun to pay attention to the impact of stress on microbial communities and the subsequent impact on health [28]. This study revealed that CRS induces dysbiosis of the oral microbiota, which subsequently exerts significant impacts on both oral and systemic health. A normal oral microbiota is crucial to maintaining oral health, and the imbalance of oral microbiota is closely related to the occurrence and development of various oral diseases, including periodontitis [29, 30]. In this study, the direct promotion of the oral microbiota dysbiosis induced by CRS in periodontitis progression was confirmed in GF mice by oral microbiota transplantation. These findings highlight the significant impact of the interaction between oral microbiome and host stress state on the development of periodontal disease.

Furthermore, our metabolomic analysis revealed that oral microbiota dysbiosis alters host metabolism in GF mice with periodontal ligation, particularly manifesting as a significant decrease in plasma EPA levels. EPA is an important Omega‐3 polyunsaturated fatty acid with multiple physiological effects on human health, which can reduce the inflammatory response and alleviate the symptoms of inflammatory diseases [31, 32, 33]. In addition, EPA modulates immune cell activity and quantity to enhance immune regulatory function, while also exerting protective effects on the nervous system [34, 35]. This study demonstrated that EPA supplementation attenuated CRS‐induced periodontitis, suggesting a protective role against oral inflammation. EPA can reduce the severity of periodontitis by lowering levels of inflammatory cytokines and reducing the release of inflammatory mediators. Given EPA's anti‐inflammatory properties, it is important to note that macrophages, a crucial type of immune cell, play a significant role in inflammatory diseases like periodontitis [36]. They are able to polarize to different effector types and thus perform different functions in the inflammatory process [37]. M1 macrophages are commonly considered to be pro‐inflammatory, while M2 macrophages are considered to be anti‐inflammatory or reparative [38]. Macrophages play a crucial role in the development of periodontitis [39]. In this study, EPA supplementation significantly attenuated M1 macrophage polarization and invasion in murine periodontal tissues, concomitant with reduced secretion of pro‐inflammatory cytokines. This regulatory effect is conducive to the regression of inflammation and tissue repair [31, 40, 41, 42]. The invasiveness of macrophages is closely linked to their roles in tumor development, metastasis, and inflammatory diseases [43]. EPA may regulate macrophage polarization and invasiveness by targeting specific signaling pathways. In this study, transcriptome sequencing was used to explore the effects of EPA on macrophages, and we found that EPA could inhibit the NF‐κB pathway. In addition, the NF‐κB pathway agonist P65a was found to reverse the effects of EPA on macrophage polarization. These findings suggest that inhibition of NF‐κB pathway may be an important mechanism by which EPA regulates macrophage polarization. The NF‐κB pathway plays a central role in the regulation of inflammation and immune response, and the activation of this pathway can promote the expression of various inflammatory factors and cytokines and exacerbate the inflammatory response [44, 45].

While the current study presents relevant findings, it is crucial to acknowledge its limitations. Specifically, the use of animal models to infer human conditions may fail to fully reflect the complex interactions between chronic stress and periodontitis in humans. In addition, the study's focus on a specific demographic for clinical data may limit the generalizability of the findings across diverse populations. The results from our clinical cohort may be more representative of or applicable to female patients. Future research should aim to address these limitations by incorporating broader human studies and integrating additional stressors to better understand the multifaceted relationship between CRS and periodontitis.

## 4 Conclusion

In conclusion, this study indicated that CRS may cause and exacerbate periodontitis by inducing oral microbiota dysbiosis, which affects metabolite levels, especially EPA. EPA attenuated periodontal inflammation by suppressing M1 macrophage polarization through inhibition of the NF‐κB signaling pathway. Our research demonstrates that the oral microbiota‐EPA‐NF‐κB pathway nexus might serve as a novel therapeutic target for stress‐induced periodontitis therapy.

## 5 Experimental Methods

### 5.1 Study Cohorts

All participants were newly diagnosed with periodontal disease at our hospital from January 2022 to March 2023. The following inclusion and exclusion criteria were used to enroll participants. Inclusion criteria: (1) Initial diagnosis of periodontitis confirmed by a periodontal specialist; (2) Age between 30 and 60 years. Exclusion criteria were as follows: (1) Individuals with fewer than 20 natural teeth; (2) Those who have undergone periodontal treatment within the preceding three months; (3) Individuals presenting with other evident oral pathologies; (4) Individuals diagnosed with infectious, systemic, neurological, or psychiatric conditions; (5) Patients who have been administered glucocorticoids or non‐steroidal anti‐inflammatory drugs in the week prior to the study; (6) Participants who have utilized antibiotics or mouthwash within the last seven days.

Relevant information about periodontal conditions and depressive symptoms was recorded, and saliva was collected from participants. Two experienced dentists conducted thorough evaluations of the periodontal status of participants using the Community Periodontal Index (CPI). In adherence to the classification framework for periodontal conditions outlined by the American Academy of Periodontology, the participants were classified into two distinct groups: mild and moderate/severe periodontitis [46]. Two psychiatrists assessed depressive symptoms using the 17‐item Chinese version of the Hamilton Depression Scale (HAMD). HAMD scores of 0–7 is defined as normal, 8–16 as mild, 17–23 as moderate, and over 24 as severe [47]. Here, patients with periodontitis are categorized as follows: those with HAMD ≥ 8 constitute the periodontitis with depressive symptoms group (PD+DS group), while those with HAMD ≤ 7 constitute the periodontitis group (PD group). Unstimulated saliva was collected from the participants using a free‐flow method [48]. The saliva was gathered between 9 and 11 a.m. No food or beverages were consumed in the 2 h preceding saliva collection. After collection, the saliva was immediately kept on ice. It was then centrifuged within 30 min at 4°C (3000 × g for 10 min), and the supernatant was stored at −80°C for future use.

### 5.2 Animals

Specific‐pathogen‐free (SPF) female Kunming (KM) mice, aged 4–6 weeks, were obtained from Jiangsu Cavensbiogle Model Animal Research Co., Ltd. The mice were housed under SPF conditions at the animal facility of the Chongqing Key Laboratory of Oral Diseases. Germ‐free (GF) female Kunming mice, aged 4–6 weeks, were housed in a flexible membrane sterile isolation chamber to maintain a controlled and contamination‐free environment. All procedures involving the GF mice, as well as the mice themselves, were managed within the NHC Key Laboratory for the Diagnosis and Treatment of Brain Function Disorders, ensuring adherence to stringent experimental standards. A minimum two‐week acclimatization period was provided for all animals, including both SPF and GF Kunming (KM) mice, prior to the initiation of any experimental procedures, such as chronic restraint stress (CRS).

Additional materials and methods are provided in the online Supporting Information.

## Author Contributions

**S.L**. and **F.L**. contributed to conceptualization, data curation, methodology development, and preparation of the original draft. **P.Y**., **Y.Z**., **L.Y**., and **Y.D**. were responsible for data curation, formal analysis, and manuscript review and editing. H.W., **Y.L**., and **J.P**. participated in conceptualization, investigation, and formal analysis. **B.Y**., **R.D.C**., **P.X**., and **P.J**. contributed to project administration, manuscript review, and editing. **X.J**. was involved in conceptualization, formal analysis, data curation, project administration, funding acquisition, and manuscript review and editing.

## Funding

This work was supported by grants from the National Natural Science Foundation of China (No. 82370968), the Chongqing medical scientific research project (Joint project of the Chongqing Health Commission and the Science and Technology Bureau) (No. 2026MSXM055), the Natural Science Foundation of Chongqing (No. CSTB2022NSCQ‐MSX1148), and Project of Luzhou Science and Technology Bureau (Grant No. 2025JYJ099).

## Ethical Approval

The clinical studies received ethical approval from the Stomatology Hospital of Chongqing Medical University Ethics Committee (Ethics number: NO. 2021–4). The animal experiments were approved by the Experimental Animal Ethics Committee of Chongqing Medical University (Approval number: NO. 2021063).

## Conflicts of Interest

The authors declare no conflicts of interest.

## Supporting information




**Supporting File**: advs74729‐sup‐0001‐SuppMat.docx.

## Data Availability

The data that support the findings of this study are available from the corresponding author upon reasonable request.
