# Supplementary material for: Dysregulation of Oral Microbial Eicosapentaenoic Acid Induced by Chronic Restraint Stress Exacerbates Periodontitis via M1 Macrophage Polarization
Source: Adv Sci (Weinh). 2026 Mar 5;13(28):e21346. doi: 10.1002/advs.202521346 (PMC13185851; doi:10.1002/advs.202521346)
Supplement: Supplementary file 1 — Supporting File: advs74729‐sup‐0001‐SuppMat.docx. [file ADVS-13-e21346-s001.docx]

Supporting information

**Dysregulation of Oral Microbial Eicosapentaenoic Acid Induced by** **Chronic Restraint Stress** **Exacerbates Periodontitis via M1 Macrophage Polarization**

Shihong Luo, Fangzhi Lou, Peiran Yang, Yu Zhang, Li Yan, Yunmei Dong, Bing Yang, Haiyang Wang, Yiyun Liu, Juncai Pu, Richard D Cannon, Peng Xie, Ping Ji, Xin Jin ^*^

**Supporting methods**

**Chronic restraint stress mouse model**

Mice in the CRS group were confined in a well-ventilated 50 mL conical tube for 2 to 5 h each day. Both the CRS and control (Con) groups of mice experienced food and water deprivation during the confinement stress period. The restraint stress procedure continued for four weeks. Following this period, oral microbiological samples were obtained from both the Con and CRS mice using sterile swabs. After swabbing, the mice were allowed a minimum of 1h to acclimatize in a quiet test chamber before behavioral evaluations were undertaken. These assessments included the tail suspension test (TST) and forced swim test (FST). In the TST, black string was tied to the tails of mice leaving a 2 cm gap from the tip of their tails. They were then suspended in an inverted position from a small metal hook for 5 min. The duration of immobility was used to assess depression-like behavior. In the FST, mice were individually introduced into a glass cylinder measuring 30 cm in height and 15 cm in diameter. The cylinder contained 18 cm of water at 23±2 °C, and the mice were maintained in this environment for 5 min. The duration of immobility was employed to gauge depression-like behavior. All behavioral tests were recorded using a video-tracking apparatus (SMART, Barcelona, Spain) and analyzed with EthoVision XT 13.0 software.

**Periodontal ligation-induced periodontitis mouse model**

The periodontal ligation procedure was conducted four weeks after the initiation of chronic restraint stress (CRS). Under isoflurane anesthesia (3% induction, 1.5% maintenance), subgingival ligation of the maxillary second molars of mice was performed with 5-0 sterile nylon sutures using a surgical microscope (Leica M320). Ligature retention was verified every 48h by dental probe assessment; dislodged sutures were replaced within 24h. The ligation was maintained for 21 consecutive days. The mice were euthanized three weeks post-periodontal ligation. Blood samples were collected immediately before euthanasia, while maxilla, teeth, and periodontal tissue samples were harvested promptly after death.

**Oral microbiome transplantation experiment**

Oral microbiome transplantation was conducted as previously described^[1]^. Fresh oral microbiome was prepared daily for transplantation over a three-day period. Oral swabs of mice were used to obtain oral microbiome for transplantation. This entailed brushing the mucosal surface within the mouse's mouth followed by the suspension of the sample in sterile saline. The suspension was centrifuged at 3000 x g for 5 min and the pellet of oral bacteria was resuspended in 2% methylcellulose. GF mice were administered 150 μL of the oral bacterial sample (5 x 10^6^ CFU/mouse) derived from either conventional mice subjected to CRS or control mice. Oral bacterial samples from the same cohort of donor mice were combined to ensure that GF mice received comparable inoculations. The GF mice underwent periodontal ligation to induce periodontitis. The mice were euthanized three weeks after the procedure, with blood samples collected immediately prior to euthanasia, and maxilla, teeth, and periodontal tissue samples harvested promptly following death.

**16S ribosomal RNA sequencing**

16S rRNA gene sequence analysis was undertaken as previously described^[1-3]^. Genomic DNA extraction from saliva and oral swab specimens was carried out using the MoBio PowerSoil DNA Isolation Kit (Carlsbad, California, USA). Preparation of the DNA library and 16S rRNA gene sequencing were undertaken by Majorbio Bio-pharm Technology Co., Ltd (Shanghai, China). The V3-V4 regions of the 16S rRNA genes were amplified utilizing universal primers (338F 5'- ACTCCTACGGGAGGCAGCAG-3' and 806R 5'- GGACTACHVGGGTWTCTAAT-3') in conjunction with adapter and barcode sequences. High-throughput sequencing of bacterial rRNA genes was conducted using the Illumina NovaSeq 6000 platform. The 16S rRNA gene sequence data were quality-filtered and analyzed with QIIME2 software (version 2020.6.0). The paired-end reads were assembled based on their overlapping relationships, and following quality control resulted in refined data. Subsequently, the data were processed using the DADA2 method for denoising to obtain Amplicon Sequence Variant (ASV) representative sequences and abundance information. Based on ASVs, a variety of statistical and visual analyses were executed, including taxonomic classification, assessment of community diversity, differential species analysis, and association analysis. The ANOISM analysis was employed to assess the variations in species complexity between the samples.

**Liquid chromatography-mass spectrometry (LC-MS/MS)**

The extraction of metabolites from plasma samples, untargeted LC-MS/MS analysis, and data preprocessing and annotation were carried out by Major Bio-pharm Technology Co., Ltd (Shanghai, China), adhering to previously published protocols^[1-3]^. For metabolite extraction, a 100 μL plasma sample was combined with 400 μL of extraction solvent (acetonitrile and methanol in equal proportions). The mixture was then subjected to ultrasonic agitation in an ice bath for 10 min and incubated at -40 ℃ for 1 h to precipitate protein. The sample was centrifuged at 2500 x g for 15 min at 4 ℃ and the resulting supernatants were retained for analysis. To construct the quality control (QC) sample, equal volumes of supernatant from each individual sample were combined. LC-MS/MS analysis was conducted using an UHPLC system (Agilent Technologies, Santa Clara, CA) coupled with a UPLC BEH Amide column (1.7 μm × 2.1 × 100 mm; Waters Corporation, Milford MA) connected to a Q Exactive HFX mass spectrometer (Orbitrap MS; Thermo). The QE HFX mass spectrometer acquired MS/MS spectra on an information-dependent basis. Raw data were converted to mzXML format using ProteoWizard and processed using the R package XCMS version 3.2. Metabolite annotations were performed utilizing the MS2 database BiotreeDB. The MetaboAnalyst R package was employed for the analysis of metabolomic data. Adjusted *p* values were calculated by applying the Benjamini-Hochberg procedure to correct for false discovery rate (FDR) from initial *p* values. Metabolites exhibiting significant changes met the criteria of variable importance in projection (VIP) > 1 and adjusted *p* value < 0.05.

**Micro-CT analysis**

To assess alveolar resorption in mice, micro-CT scans were utilized to examine the mouse maxilla. First, the mice were humanely sacrificed in accordance with ethical guidelines, and the jaws with the surrounding intact soft tissues were carefully dissected. The samples were then fixed in 4% paraformaldehyde at 4°C for 24 - 48 hours. After fixation, they were transferred to 70% ethanol for storage until the scanning process. A high - resolution micro-CT system (Skyscan 1176) was employed for the scanning. The specific scanning parameters were set as follows: a voxel size of 10μm, an X - ray voltage of 50 kV, and an X - ray current of 300 μA. Finally, the proprietary software (NRecon for Skyscan) was used to reconstruct 3D volumes from the raw projections, enabling a detailed analysis of the alveolar resorption in the mouse maxilla.

**TRAP staining**

To identify and quantify osteoclasts in alveolar bone, tartrate-resistant acid phosphatase (TRAP) staining was conducted on periodontal tissue utilizing a TRAP staining kit (Solebel, China). Samples were incubated in a 4% polyformaldehyde solution for 24 h, followed by decalcification; The decalcified specimens were cut into 4 μm sections using frozen blocks embedded in optimal cutting temperature compound (OCT) (Beyotime, China). The frozen sections were fixed in a 4% paraformaldehyde solution for 10 min and then rinsed with PBS to remove excess fixative. The sections were incubated in TRAP staining solution at 37℃ for 1 h. Surplus stain was removed by rinsing with PBS. The sections were counter stained with nuclear dye (methyl green) for 1 min, excess pigment was removed by rinsing with PBS and the slides sealed with a water-based sealer.

**Enzyme linked immunosorbent assay (****ELISA)**

The concentrations of IL-1β, TNF-α, CD86, and CD206 in mouse plasma, periodontal tissues and cell culture fluid were measured using an ELISA kit (Jianglai, Shanghai, China) according to the manufacturer's guidelines. The concentration of IL-6 in mouse plasma and cell culture fluid was measured using an ELISA kit (R&D Systems, Minneapolis, MN, USA) according to the manufacturer's guidelines. The absorbance readings at 450 nm were recorded using a microplate reader (SpectraMAX iD5, USA), and the sample concentrations were determined with reference to standard curves.

**Quantitative reverse-transcription PCR (****qPCR)**

Total RNA was isolated from periodontal tissues, or cells, with Trizol Reagent (Beyotime, China). Reverse transcription was carried out using the PrimeScript RT Reagent Kit with gDNA Eraser (Takara, Japan). The qPCR was carried out using the QuantStudio™ 7 Flex Real-Time PCR System (Thermo Fisher Scientific) employing TB Green® Premix DimerEraser™ (Takara, Japan). The primer sequences corresponding to the genes investigated are presented in sTable 1. The relative mRNA expression was determined using the 2^−ΔΔCT^ method and normalized against the reference gene GAPDH.

**Measuring the effect of EPA and p65a on periodontitis under CRS conditions**

To investigate the effects of the microbial metabolite EPA on periodontitis under CRS conditions, all mice were subjected to a two-week antibiotic cocktail (ABX) treatment consisting of 1 g/L ampicillin (SY007, Beyotime, China), 1 g/L metronidazole (HY-B0318, MedChemExpress, Monmouth Junction, NJ, USA), 1 g/L neomycin (ST2533, Beyotime, China), and 0.5 g/L vancomycin (ST2807, Beyotime, China), followed by a 4-week chronic stress procedure. Subsequently, periodontal ligation and EPA or p65a treatment were initiated. EPA (HY-B0660, MCE) was administered orally at 50 mg/kg every other day for three weeks. Similarly, p65a (HY-134476, MCE) was administered via intraperitoneal injection at a dose of 5 mg/kg on the same schedule. Upon completion of the experiment, the mice were euthanized, and tissue samples were collected.

**Cell line and culture**

The murine macrophage cell line Raw 264.7 (RRID: CVCL_0493) was procured from the American Type Culture Collection (ATCC, Manassas, VA, USA) and received in June 2023. Upon arrival, the cell line was authenticated by short tandem repeat (STR) profiling and confirmed to be free of mycoplasma contamination prior to its use in experiments. Raw264.7 cells were maintained in Dulbecco's modified Eagle's medium (Gibco, Carlsbad, California, USA) enriched with 10% fetal bovine serum (Invitrogen Life Technology, Carlsbad, California, USA) and 1% penicillin-streptomycin (Gibco, Carlsbad, California, USA) at 37°C with 5% CO_2_. Raw264.7 cells were stimulated with LPS at a concentration of 10 nM EPA treatment (50 μM, HY-B0660, MCE) and the NF-κB/p65 activator treatment (5 μM, HY-134476, MCE) were administered. Following a 24 h incubation under these conditions, the cells were collected for further analysis.

**Transwell migration assay**

The invasive capacity of macrophages was assessed using a transwell assay, and the influence of EPA on the invasive capacity of macrophages was analyzed. Fifty microliters of BD Matrigel matrix gel was diluted with DMEM medium at a 1:8 ratio, evenly distributed in the upper chamber of the transwell apparatus (24-well plate, aperture 8 μm), and incubated at 37°C for 30 min. RAW264.7 cells were centrifuged and re-suspended in serum-free medium, adjusting the cell density to 1 x 10^5^/mL. Two hundred microliters of serum-free cell suspension were uniformly added to the upper chamber containing the aforementioned cells, while 600 mL of complete medium was introduced into the lower chamber of the control group, and 600 mL of complete medium with 50 μM EPA was added to the lower chamber of the experimental group. The transwell plates were then incubated in 5% CO_2_ at 37℃. After 24 h of incubation, the cells were fixed for 15 min with 4% paraformaldehyde and stained for 10 min with 0.1% crystal violet; the circular matrix gel and cells were carefully removed, and the number of macrophages traversing the circular cavity was observed and quantified using an inverted microscope.

**Transcriptome sequencing**

To investigate the mechanism of EPA regulation of macrophage polarization, RNA-seq was employed to identify differential gene expression and examine pertinent pathways. Total RNA was extracted from control and EPA macrophages utilizing an RNA extraction kit (Beyotime, China); magnetic beads with Oligo dT were used to isolate mRNA from the total RNA. cDNA was synthesized from the mRNA through reverse transcription (Takara, Japan). An End Repair Mix is incorporated into the double-stranded cDNA to repair the ends, followed by the addition of one Adapter for subsequent attachment to the Y connector. cDNA fragments linked to the adapter were sorted, purified, and then amplified by PCR. The quantified PCR products were pooled in equimolar ratios, followed by bridge PCR amplification to generate clusters, and finally sequenced on the Illumina NovaSeq 6000 platform.

Quality assessment of all raw sequencing data was performed. Fastp software was used to filter the original sequencing data to obtain high-quality sequencing data (Clean data). The clean reads were aligned to the reference genome using HiSat2, and the resulting mapped data were assessed for alignment quality. RSEM software was employed for the quantitative analysis of gene expression levels. DESeq2 software was used to analyze the differences in gene expression between samples, with the criteria for differentially expressed genes (DEGs) set as: FDR < 0.05 and |log2FC| ≥ 1. Diamond and ID mapping software were utilized to categorize the genes, and Kyoto Encyclopedia of Genes and Genomes (KEGG) annotations were performed for the genes exhibiting differential expression.

**Western blot analysis**

Total cellular protein was extracted utilizing RIPA lysis buffer enriched with a protease inhibitor (Beyotime, China). Subsequently, the proteins were resolved by SDS-PAGE and transferred onto PVDF membranes (Merck Millipore, Ireland). The membranes were then blocked with 5% BSA and incubated overnight at 4℃ with primary antibodies. The primary antibodies employed included rabbit anti-GAPDH (1:1000; 2118s, Cell Signaling Technology), anti-IKK (1:1000, HY-P80414, MCE), and anti-p-NFκB (1:1000; HY-P80470, MCE). Following incubation with the primary antibodies, the membrane was washed three times with TBST. Subsequently, it was incubated with goat anti-rabbit HRP-conjugated secondary antibody (1: 10,000). Protein blots were visualized using the ECL Plus ChemiDoc™ Touch MP system (BIO-RAD, USA), with GAPDH acting as an internal control.

**Statistical analysis**

Data are presented as mean ± standard deviation (SD) and were analyzed using a two-tailed Student’s t-test or one-way ANOVA. Correlation analysis was executed employing Pearson correlation coefficients. Statistical tests were carried out using GraphPad Prism 8.0 and SPSS 25.0. Differences were deemed significant if the *p* < 0.05.

**References**

[1] Lou F, Luo S, Kang N, et al. Oral microbiota dysbiosis alters chronic restraint stress-induced depression-like behaviors by modulating host metabolism [J]. Pharmacol Res, 2024, 204(107214.

[2] Luo S, Lou F, Yan L, et al. Comprehensive analysis of the oral microbiota and metabolome change in patients of burning mouth syndrome with psychiatric symptoms [J]. J Oral Microbiol, 2024, 16(1): 2362313.

[3] Lou F, Yan L, Luo S, et al. Dysbiotic oral microbiota-derived kynurenine, induced by chronic restraint stress, promotes head and neck squamous cell carcinoma by enhancing CD8(+) T cell exhaustion [J]. Gut, 2025,

**Supporting figure and figure legend**


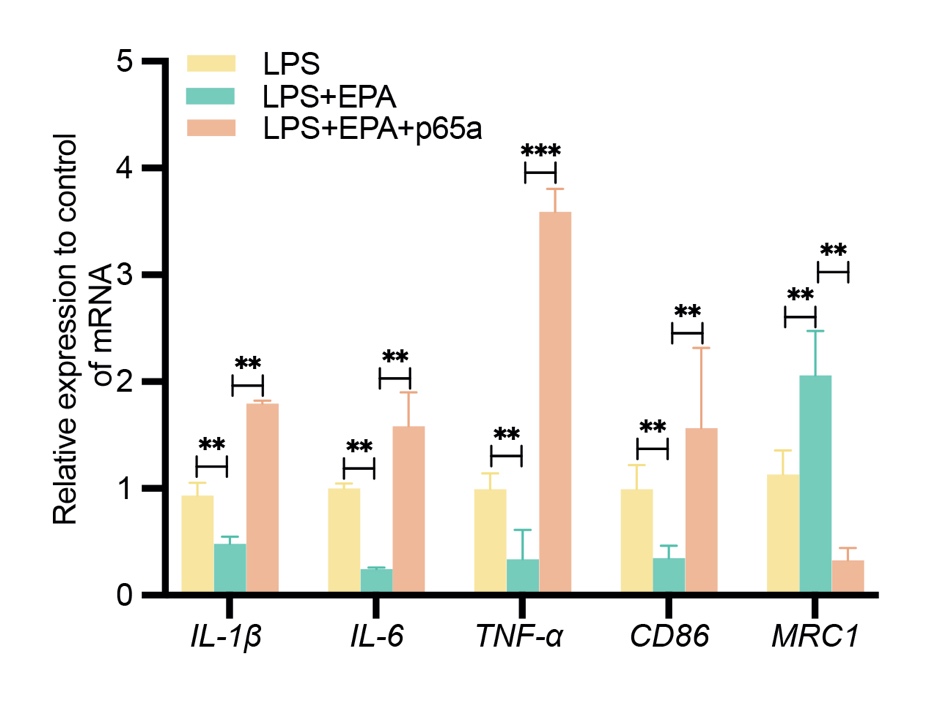


**Supporting figure 1.** mRNA expression levels of IL-1β, IL-6, TNF-α, CD86, and MRC1 in RAW264.7 cells analyzed using qPCR.

**Supporting table**

**Supporting table 1. The primer sequences employed for qPCR**

| **Gene** | **Forward 5’-3’** | **Reverse 5’-3’** |
| --- | --- | --- |
| *IL-β* | AGATGCAATCCTTGATAACCTG | CTGCTTCCTAACTTCATGCTC |
| *IL-6* | TGGGACCATCATAACATCACATC | GGATGACAATGTACGCTTCG |
| *TNF-α* | TGCTAAAAGGATTCAAGGCTG | AACTCCTCACTGCGGTTCTGG |
| *CD86* | GCTGCTCCTACTGTTTACTACG | CTGCTCAATTTAAAGTCCTG |
| *MRC1* | GCCATCATCGCTATCCTTCTG | CGCCGTTTCATCCATACCAC |
| *GAPDH* | CAGGGCTATCTTGGGCTACAC | GTTGCTGTTGACAGTCGCAGG |
